# Parastomal Hernia Repair Following Ileal Conduit Urinary Diversion Using the Sugarbaker Technique With Biological Mesh: A Case Report

**DOI:** 10.7759/cureus.82576

**Published:** 2025-04-19

**Authors:** Catarina R Santos, Ligia Santos, Luís Sousa, Zara Caetano

**Affiliations:** 1 General Surgery, Centro Hospitalar Barreiro Montijo, Barreiro, PRT

**Keywords:** abdominal wall reconstruction, biological mesh, ileal conduit urinary diversion, parastomal hernia, sugarbaker technique

## Abstract

Parastomal hernia is a rare but significant complication following ileal conduit urinary diversion, often leading to impaired stoma function and patient discomfort. Surgical repair remains challenging due to anatomical constraints, particularly the posterior fixation of the conduit via the ureters, which can limit mobilization. Although traditionally deemed unsuitable for ileal conduit-related parastomal hernia, the Sugarbaker technique has demonstrated favorable outcomes in carefully selected cases. We present a case of a 66-year-old female who underwent successful parastomal hernia repair using the Sugarbaker technique with biological mesh, achieving an uneventful recovery and no recurrence at three months postoperatively. This case highlights the feasibility of this approach, contributing to the growing body of evidence supporting mesh-based techniques in complex parastomal hernia repairs.

## Introduction

Parastomal hernia (PSH) is a frequent yet often underestimated complication following stoma creation, with reported incidence rates of 48.1% after colostomy and 28.3% after ileostomy [1]. While conservative management may be appropriate in certain cases, symptomatic or complicated PSH often necessitates surgical intervention. However, recurrence rates remain high, and no consensus exists regarding the optimal repair technique [2,3]. Among available techniques, the keyhole and Sugarbaker repairs are widely utilized [4]. The keyhole technique involves placing an underlay mesh with a central opening through which the bowel is passed. The Sugarbaker technique, originally introduced in 1985, lateralizes the bowel and covers it with intraperitoneal mesh to reduce recurrence risk [5]. Despite its success in colostomy-related PSH, Sugarbaker repair has been considered unsuitable for ileal conduit hernias due to the posterior fixation of the conduit via the ureters, which may limit mobility and increase surgical complexity [6]. Recent studies, however, suggest that Sugarbaker repair can be successfully applied in ileal conduit PSH with low recurrence rates [7]. Moreover, advancements in mesh technology, including biological and biosynthetic meshes, have allowed for improved outcomes, particularly in contaminated surgical fields [8,9]. Additionally, epidemiological studies highlight the increasing burden of hernia repairs, reinforcing the need for evidence-based surgical techniques to optimize patient outcomes [10]. Here, we present a case of a PSH following ileal conduit urinary diversion, successfully repaired using the Sugarbaker technique with biological mesh, demonstrating its feasibility in carefully selected patients.

## Case presentation

**Figure 1:**
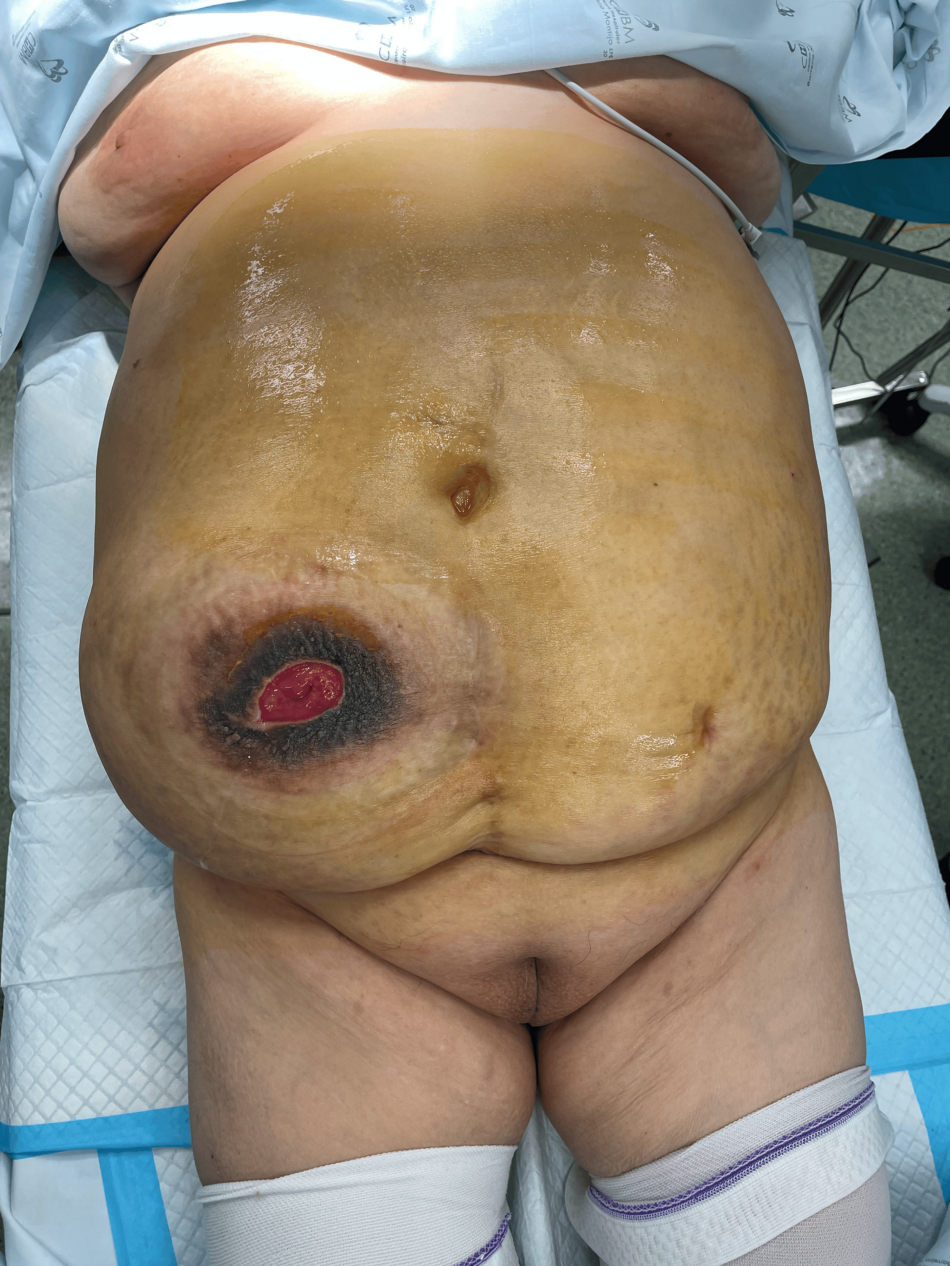
Parastomal hernia

A 66-year-old female with a history of radical cystectomy and hysterectomy with anexectomy underwent ileal conduit urinary diversion eight years ago due to bladder carcinoma. Her comorbidities included type II diabetes mellitus, hypertension, and hypercholesterolemia. She was referred for a general surgery consultation due to a progressively enlarging PSH, causing discomfort and interfering with the ostomy appliance function. On physical examination, the patient was in good general condition, with a soft, non-tender abdomen. A large, reducible PSH was noted (Figure 1).

**Figure 2:**
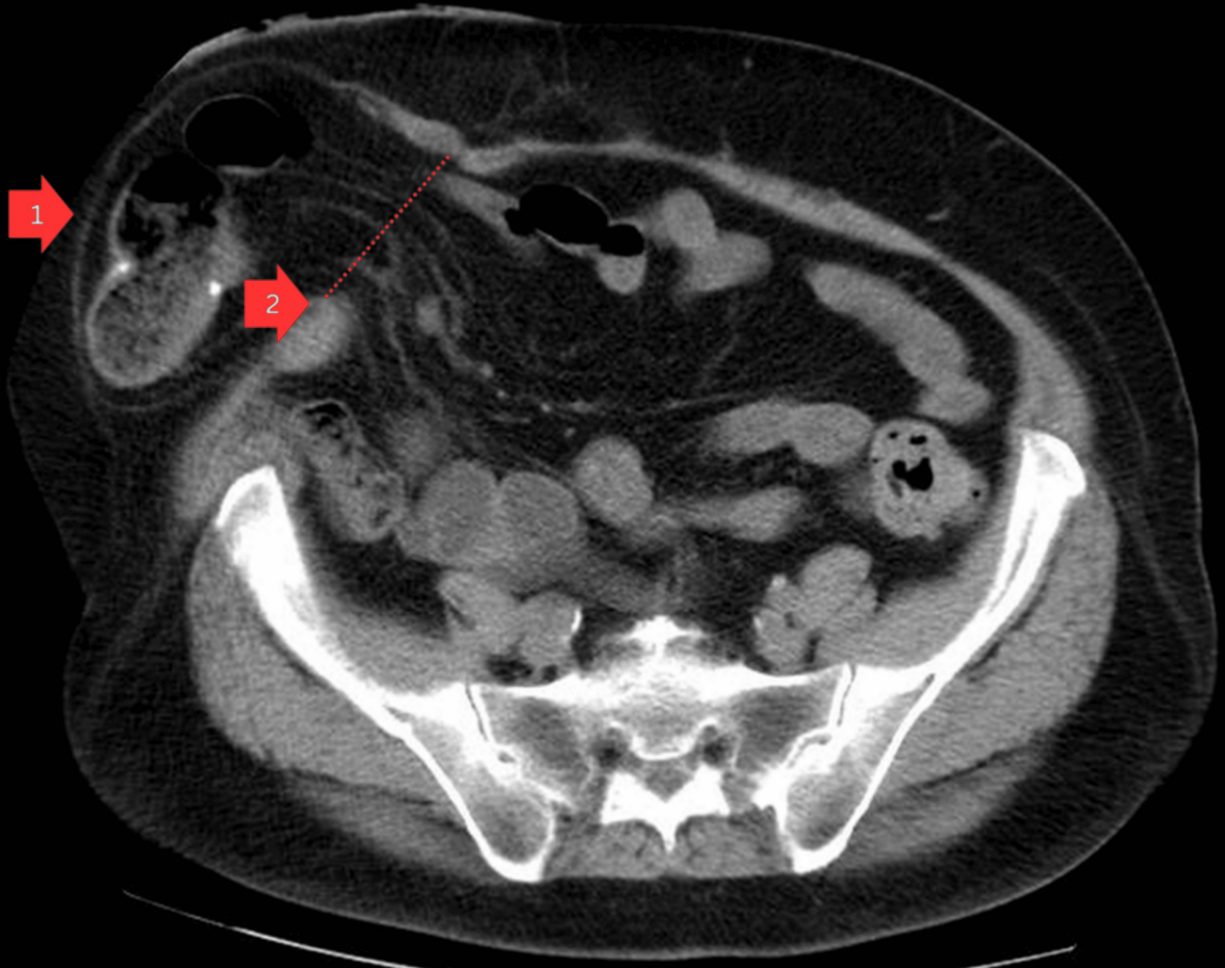
Abdominopelvic computed tomography axial imaging identifying parastomal hernia Arrow 1: hernial sac, Arrow 2: hernial orifice

Abdominopelvic computed tomography imaging confirmed a 9 cm PSH orifice, containing small bowel loops (Figure 2).

**Figure 3:**
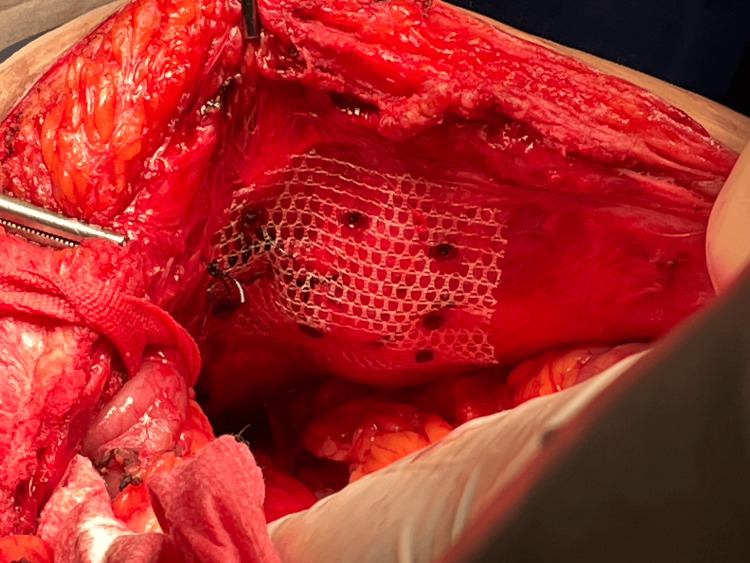
Polypropylene mesh covering the defect

Given the hernia’s size and symptoms, surgical repair was indicated. The patient underwent hernia repair using the Sugarbaker technique with biological mesh (sterile, acellular type I collagen matrix derived from porcine skin). A midline relaparotomy was performed, excising the previous scar. Adhesiolysis was carried out to release omental and bowel adhesions to the ileal conduit. The hernia orifice was isolated, and an integrity test using methylene blue confirmed no leaks. The hernia defect was closed using simple separated long-term absorbable synthetic monofilament sutures, and a flat polypropylene mesh was placed over the defect, secured with tacks (Figure 3).

**Figure 4:**
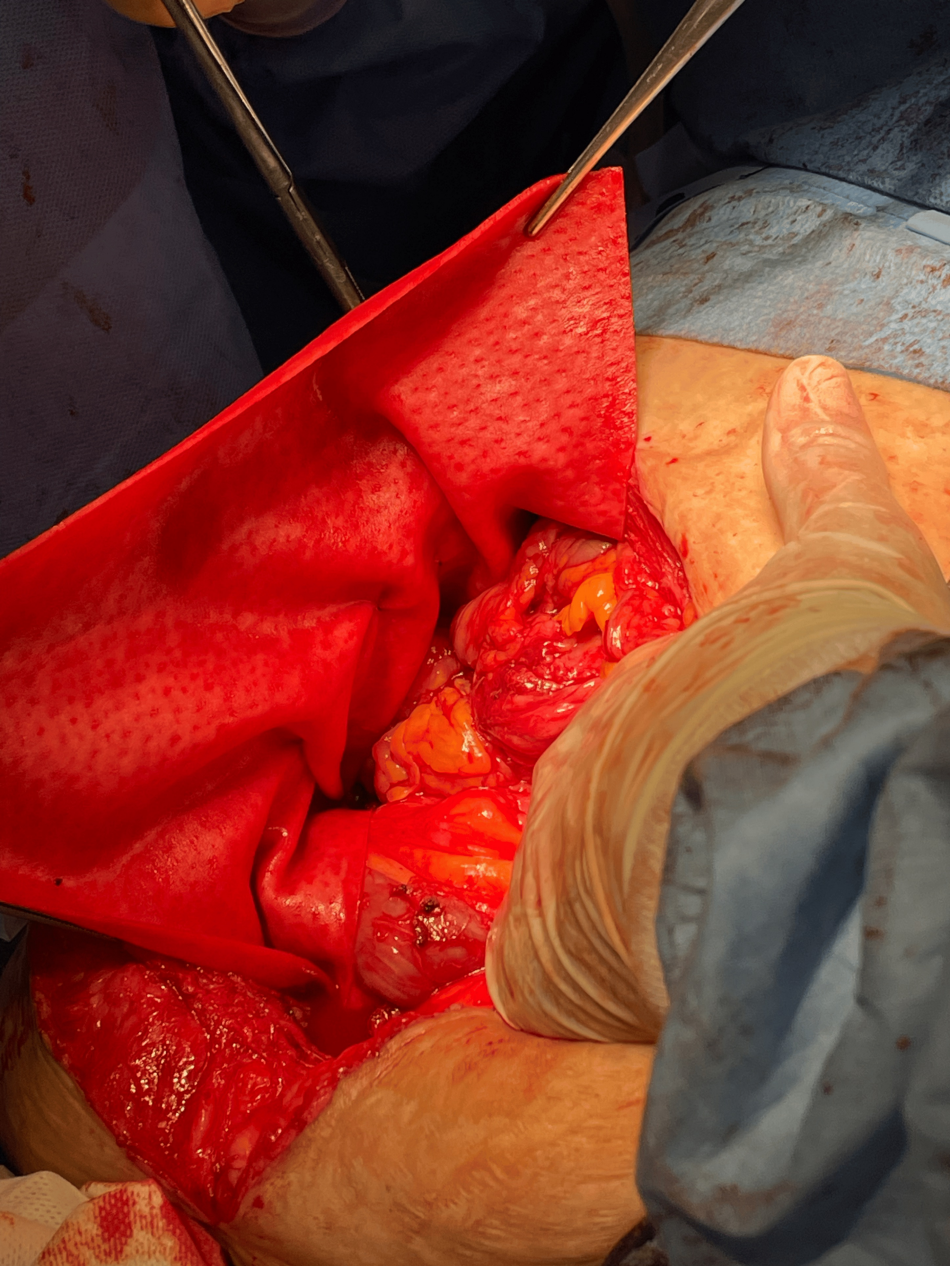
Sugarbaker technique with biological mesh

A biological mesh was applied peristomally using the Sugarbaker technique, fixated to the right lateral abdominal wall with long-term absorbable synthetic monofilament suture. The biological mesh was placed over the polypropylene mesh, acting as a protective barrier to reduce adhesion-related complications with the intestine (Figure 4).

The left-sided mesh fixation reinforced the aponeurotic closure. Aspiration drainage was placed in the subaponeurotic space, exteriorized through a paramedian counter-incision. The aponeurotic plane was closed with long-term absorbable synthetic monofilament suture, and the skin was closed with non-absorbable suture and staples.

The postoperative course was uneventful, and the patient was discharged on postoperative day 4 with the drain in place. One week postoperatively, she was asymptomatic, with no abdominal pain and a functional ileal conduit. The surgical wound was intact, and the drain was removed at two weeks. At six months of follow-up, the patient remained asymptomatic with no evidence of recurrence.

## Discussion

Despite advancements in mesh-based PSH repair, recurrence remains a challenge [11]. The keyhole and Sugarbaker techniques are two widely used methods for PSH repair. The keyhole technique, which involves creating an opening in the mesh for the stoma to pass through, is generally considered when the bowel cannot be easily lateralized; however, it carries a higher risk of recurrence due to potential mesh separation or enlargement of the keyhole opening. The Sugarbaker technique, which lateralizes the bowel and overlays the mesh without an aperture, has demonstrated lower recurrence rates and is often preferred, especially in complex or recurrent hernias and in cases involving an ileal conduit, as in our patient. In our case, the Sugarbaker approach was selected for its durability and favorable outcomes. While the Sugarbaker method has historically been avoided in ileal conduit hernias due to concerns over conduit immobility and ureteral kinking [12], recent studies support its use in this context, reporting a low recurrence rate of 10% and no increased complication rate compared to the keyhole technique [9]. The use of biological mesh, following the application of polypropylene mesh to reinforce the closure of the hernia defect, ensured a reliable technique to minimize the risk of infection and recurrence [13]. Further research comparing synthetic vs. biologic mesh, laparoscopic vs. open approaches, and long-term recurrence rates is necessary to refine surgical decision-making and optimize patient outcomes.

A 2023 multicenter study compared outcomes between biologic and synthetic meshes in PSH repairs, concluding that biologic mesh was associated with significantly fewer surgical site infections, particularly in high-risk or contaminated fields [2]. This supports the choice of biologic mesh in our case, especially given the proximity of the ileal conduit and potential for contamination. The study further emphasized that recurrence rates were not statistically different between mesh types, reinforcing the importance of mesh selection based on patient-specific factors.

Moreover, emerging evidence suggests that hybrid techniques incorporating both Sugarbaker principles and retromuscular positioning of mesh can further optimize outcomes. A 2022 review highlighted that these modifications may enhance mesh integration and reduce both recurrence and complications, particularly in anatomically challenging cases [14]. These insights encourage further adaptation of the Sugarbaker technique, particularly in complex scenarios like ileal conduit-associated PSH, and reflect the growing sophistication in abdominal wall reconstruction strategies.

Hydronephrosis from ureteral kinking is a known risk after PSH repair with the Sugarbaker technique, especially in ileal conduit cases [15,16]. We monitored renal function using serial serum creatinine, which remained stable, with no signs of hydronephrosis.

While a laparoscopic approach is a recognized option for PSH repair, we elected to perform an open abdominal approach in this case due to several important considerations. The patient had a complex surgical history, including radical cystectomy and hysterectomy with anexectomy, followed by ileal conduit urinary diversion. This significantly increased the likelihood of dense intra-abdominal adhesions and distorted pelvic anatomy, raising the risk of bowel or conduit injury during laparoscopic access and dissection. Moreover, our surgical team had limited experience with laparoscopic repair in the specific context of ileal conduit PSHs, and we determined that an open approach would offer the greatest margin of safety and the most favorable outcome. This strategy enabled meticulous adhesiolysis, careful handling of the urinary conduit, and secure mesh placement using the Sugarbaker technique, resulting in a durable and complication-free repair.

This case demonstrates the successful repair of an ileal conduit PSH using the Sugarbaker technique with biological mesh. Despite concerns regarding ureteral fixation, the patient recovered uneventfully with no recurrence at three months, suggesting that this approach is a viable option in selected cases. The findings are particularly relevant to general and colorectal surgeons, with broader implications across urology and abdominal wall reconstruction. As surgical techniques and biomaterials continue to evolve, further research is needed to establish the optimal approach to ileal conduit PSH repair.

## Conclusions

This case highlights the successful repair of an ileal conduit PSH using the Sugarbaker technique with biological mesh. Despite theoretical concerns regarding ureteral fixation, the patient recovered uneventfully, with no recurrence of herniation observed at three months postoperatively. Given the risk of ureteral obstruction in such repairs, we incorporated postoperative surveillance with serial serum creatinine measurements, which remained stable and within normal limits-suggesting preserved renal function and no evidence of hydronephrosis. These findings support the viability of this approach in carefully selected cases.

While this case demonstrated a successful outcome at three months postoperatively with no evidence of recurrence or infection, it is important to recognize that hernia recurrence often occurs beyond this early postoperative window-sometimes months or even years after repair. Surgical site infections, by contrast, typically present within the first 30 days, though mesh-related infections may emerge later. As such, long-term follow-up is essential for accurately assessing the durability and safety of any PSH repair technique. The case is particularly relevant to general and colorectal surgeons and has broader implications for urologists and those involved in abdominal wall reconstruction. As surgical techniques and biomaterials continue to advance, further research is warranted to define best practices and optimize outcomes in ileal conduit PSH repair.
